# Core community structure recovery and phase transition detection in temporally evolving networks

**DOI:** 10.1038/s41598-018-29964-9

**Published:** 2018-08-28

**Authors:** Wei Bao, George Michailidis

**Affiliations:** 1 Department of Physics, University of Michigan, Ann Arbor, USA; 2 Department of Statistics and the Informatics Institute, University of Florida, Gainesville, USA

## Abstract

Community detection in time series networks represents a timely and significant research topic due to its applications in a broad range of scientific fields, including biology, social sciences and engineering. In this work, we introduce methodology to address this problem, based on a decomposition of the network adjacency matrices into low-rank components that capture the community structure and sparse & dense noise perturbation components. It is further assumed that the low-rank structure exhibits sharp changes (phase transitions) at certain epochs that our methodology successfully detects and identifies. The latter is achieved by averaging the low-rank component over time windows, which in turn enables us to precisely select the correct rank and monitor its evolution over time and thus identify the phase transition epochs. The methodology is illustrated on both synthetic networks generated by various network formation models, as well as the Kuramoto model of coupled oscillators and on real data reflecting the US Senate’s voting record from 1979–2014. In the latter application, we identify that party polarization exhibited a sharp change and increased after 1993, a finding broadly concordant with the political science literature on the subject.

## Introduction

There has been a lot of work across different scientific communities including computer science, applied physics, statistics and the social sciences in developing methods for the analysis of network data^1^. The impetus for these developments has been the availability of new data in biology (e.g. protein-protein interactions, product-substrate relationships amongst compounds, or ecological communities of commensal, symbiotic and pathogenic microorganisms), friendship relationships in social media platforms such as Facebook, Instagram and Twitter, transactional data between consumers or business organizations, just to name a few^2–6^. Such data capture interactions between a set of entities (e.g. biomolecules, physical persons, companies) giving rise to a network structure.

A wide range of topics can be studied on networks, including constructing representations, effective visualization of their structure, descriptive analysis of their characteristics, study of network formation models and their behavior and study of dynamically evolving phenomena (like epidemics or information diffusion) on networks^1,7^. A topic that has recently attracted a lot of interest is that of identifying community structure in observed networks, as well as developing network formation models that exhibit such structure. A community on a network is heuristically defined as a set of nodes exhibiting high degree of interconnectivity in comparison to other nodes in the network. Its importance stems from the fact that it represents a defining characteristic of real world networks; for example, sets of close friends give rise to such communities in social networks, or sets of closely interacting biomolecules (functional pathways) in biological networks. A large number of fast and efficient algorithms have been proposed in the literature to identify such communities in networks, including minimum cut based graph partitioning, hierarchical clustering, k-means based clustering and spectral clustering (for a comprehensive review, see^8^ and references therein). Another class of algorithms is based on maximization of a quality function known as “modularity”.

〈**Footnote:** It is a function defined as^9^:

1 $$Q=\frac{1}{2m}\,\sum _{ij}\,({A}_{ij}-\frac{{k}_{i}{k}_{j}}{2m})\delta ({g}_{i},{g}_{j})$$

where *A* is the adjacency matrix, *g*_*i*_ stands for the community to which node *i* belongs, ${k}_{i}={\sum }_{j}\,{A}_{ij}$, $2m={\sum }_{ij}\,{A}_{ij}$ and *δ*(*g*_*i*_, *g*_*j*_) = 1 if *g*_*i*_ = *g*_*j*_ and 0 otherwise. A higher modularity value indicates a better partition for the network under consideration〉.

Over possible partitions of the network; given the computational complexity of this optimization problem various greedy variants, as well as algorithms based on simulated annealing and spectral optimization^10–13^ have been developed. Finally, on the network formation models front exhibiting community structure, the stochastic block model (SBM) and its variants (e.g. degree corrected SBM) have been the objects of intensive study^14–16^.

However, most of the focus to date has been on static networks, where a single snapshot is available. In many applications, one has access to a sequence of network snapshots evolving over time. It is then of great interest to identify communities in such dynamically evolving networks and also investigate whether their structure remains fixed or exhibits changes over time. There has been some recent work on this subject. For example^17^ developed a generalized modularity quality function that reflects the temporal dynamics of a sequence of networks, while^18^ introduced the concept of common communities and proposed a method to detect them in a sequence of networks by optimizing an objective function based on a node-wise membership matrix. Further^19^, developed a robust community detection algorithm for this problem, by optimizing a quality function (e.g. modularity) based on null statistical models assumed to generate the network data; examples of such models include the Newman-Girvan one and correlation/similarity ones^9,19,20^.

However, an issue not adequately explored in the literature is the identification of time epochs where significant changes (phase transitions) occur in the network structure and also the community structure between them. Some previous work includes^18^ and^21^, the latter using an Ising model and assuming the existence of a single change in the network structure over time. Nevertheless, such changes are common in many applications. A recent example comes from the change in the connectivity patterns in networks of asset returns before, during and after the financial crisis of 2008 (for details see^22^), while another one relates to changes in brain connectivity before and after epileptic seizures^23^. A third example stems from changing patterns of political polarization amongst US legislators, which is examined later on in this study.

Our proposed modeling framework for the problem at hand assumes that given a sequence of *T* network snapshots, the community structure exhibits significant changes (phase transition) at time periods ${\{[{\tau }_{m}^{-},{\tau }_{m}^{+}]\}}_{m=1}^{M}$, while it remains invariant within time segments $({\tau }_{m}^{+},{\tau }_{m+1}^{-})$, where ${\tau }_{m}^{-}$ and ${\tau }_{m}^{+}$ represents the start and end time points of the *m*-th phase transition epoch, respectively. Possible changes during phase transitions include merging or division of existing communities, growth by adding members to them or their extinction altogether, while during stable periods the community can exhibit perturbations in its structure; either *dense* ones that involve a number of nodes in the community, but small in magnitude in the sense that the strength of the links between nodes changes by a small amount, or *sparse* ones that involve isolated nodes, but the strength of the corresponding links to selected other nodes can be large in magnitude. Technically, we assume that the community structure can be captured by a *low-rank* weighted adjacency matrix, while the perturbations correspond either to the addition of small magnitude dense components or large magnitude sparse components.

## Results

### Model formulation and Optimization Strategy

Consider a sequence of *T* weighted adjacency matrices ${\{A(t)\}}_{t=1}^{T}$ that encaptulate the structure of a network comprising of *n* nodes and their corresponding edges. It is a symmetric matrix and the edge magnitude ${A}_{i,j}(t),i,j=1,\ldots ,n$ capture the strength of association between nodes *i* and *j*. In the simplest case, ${A}_{ij}(t)\in \{0,1\}$ indicate whether nodes *i* and *j* are connected or not.

It is assumed that *A*(*t*) can be decomposed as follows: *A*(*t*) = *L*(*t*) + *S*(*t*) + *E*(*t*), where *L*(*t*) is a low-rank matrix, *S*(*t*) is a sparse one with most of its elements being zero and *E*(*t*) a dense matrix with $\parallel E(t){\parallel }_{F} < \varepsilon$ for some small *ε* > 0 and where $\parallel \cdot {\parallel }_{F}$ denotes the Frobenius norm. This model captures the presence of community structure in networks through the low-rank component, as well as possible small sparse and/or dense perturbations as explained in the previous section. It is also compatible with the popular network formation model that gives rise to community structure, namely the Stochastic Block Model (SBM)^15^. Specifically, the SBM assumes an undirected network on *n* nodes and that the nodes are partitioned into *K* blocks. Then, edges are formed according to the following stochastic mechanism. Node *i* is connected to node *j* with an edge, whose probability of occurring only depends on the blocks (communities) to which *i* and *j* belong to. It is commonly assumed that the probabilities for edges between *i* and *j* in the same community are significantly higher than those for nodes *i* and *j* in different communities. The SBM has been the object of intense study in recent years^14–16^. It can be seen that this mechanism gives rise to a low-rank structure *L*, corrupted with noise *E*, thus captured by the posited model. In fact, the proposed model also allows for “spiky” noise in the form of the sparse matrix *S* and as already mentioned can accommodate weighted adjacency matrices as well.

In what follows, we use the time point *τ*_*m*_ to represent the *m*-th phase transition time period $[{\tau }_{m}^{-},{\tau }_{m}^{+}]$. For a sequence of adjacency matrices, we make the additional assumption that the low rank structure is invariant between phase transition time points *τ*_*m*_, while the perturbations are allowed to vary freely. This is consistent with the intuition that community structure can be *slowly evolving* over time, while individual edges (connections) between nodes can exhibit a higher degree of variability in their patterns. Hence, our modeling framework assumes that:

$$L(t)={L}_{m}I(t\in ({\tau }_{m},{\tau }_{m+1}))+S(t)+E(t),\,t=1,\ldots ,T,\,0={\tau }_{0} < {\tau }_{1} < \cdots  < {\tau }_{m} < \cdots  < {\tau }_{M}=T.$$

Note that the problem of decomposing a matrix into low-rank and sparse/dense components has been investigated in the literature, due to its relevance in matrix completion problems^24–29^ that emerged from recommender systems, compressed sensing, system identification, anomaly detection and related applications^28,30–33^. Specifically, the low rank recovery problem can be formulated as a rank minimization problem subject to certain constraints. To make the problem computationally tractable (convex), a nuclear norm.

〈**Footnote:** The nuclear norm of a matrix $X\in {{\bf{R}}}^{M\times N}$ of rank *r* is defined as the summation of all the singular values $\parallel X{\parallel }_{\ast }:\,={\sum }_{i=1}^{r}\,{\sigma }_{i}$, where *σ*_*i*_’s are the singular values of *X* and are equal to the square roots of the eigenvalues of *XX*^*T*^〉.

Was introduced in^24^. Subsequent work^25,26^ examined the following variant ${{\rm{\min }}}_{L,S}\,\parallel L{\parallel }_{\ast }+\gamma \parallel S{\parallel }_{1}$, subject to exact recovery *A* = *L* + *S*, while a further extension was studied in^27^ where the objective function remained the same, but the constraints allowed low-rank recovery subject to sparse and also dense noise *E*; i.e. *A* = *L* + *S* + *E* with $\parallel E{\parallel }_{F}\le \varepsilon ,\varepsilon  > 0$. In the above, $\parallel \cdot {\parallel }_{1}$ denotes the ${\ell }_{1}$ norm for the vectorized form of its matrix argument. Further, in theoretical work^25,27^, showed that the problem is feasible and admits a correct solution if an *incoherence condition* between *L*, *S* and *E* is satisfied, which intuitively requires that the matrices *E* and *S* can not exhibit low-rank structure.

In the presence of dense noise, the corresponding optimization problem can be formulated as

$$\begin{array}{ll}{\rm{\min }} & \parallel L{\parallel }_{\ast }+\gamma \parallel S{\parallel }_{1}+\alpha \parallel E{\parallel }_{F}^{2}\\ \,{\rm{s}}.\,{\rm{t}}. & L+S+E=A\end{array}$$

with the two tuning parameters *γ* and *α* controlling the trade-off amongst the low rank, sparse and dense noise components.

Turning our attention to the problem at hand, we can analogously formulate the problem, while we need to incorporate an additional constraint that would force recovery of the *same* low-rank (community) component between phase transition epochs. The latter task can be accomplished by adding a total variation penalty encouraging similarity between successive time estimates of *L*(*t*), given by

2 $$\begin{array}{ll}{\rm{\min }} & \sum _{t=1}^{T}\,[\parallel L(t){\parallel }_{\ast }+\gamma (t)\parallel S(t){\parallel }_{1}+\alpha (t)\parallel E(t){\parallel }_{F}^{2}]+\sum _{t=2}^{T}\,\lambda (t)[\parallel L(t)-L(t-1){\parallel }_{F}^{2}]\\ \,{\rm{s}}.\,{\rm{t}}. & A(t)=L(t)+S(t)+E(t),\,t=1\cdots T\end{array}$$

with the tuning parameters *λ*(*t*) controlling the degree of discrepancy between consecutive low rank components *L*(*t*). It is worth mentioning that the squared Frobenius norm for the total variation penalty can be replaced by other norms such as the ${\ell }_{1}$ to achieve the same objective, and the proposed strategy below would go through with minor modifications.

#### Brief Overview of Change Point Analysis Methods

The problem has been extensively studied in the statistics literature for *single time series* data. Further, an extensive body of theoretical results -convergence rates of various estimators, as well as asymptotic distributions for the change point (phase transition epoch)- have been established when a *single* change point is assumed (for a comprehensive review see^34,35^). More recently, the focus has shifted to developing fast procedures for identifying *multiple* change points in a single time series and also providing probabilistic guarantees for identifying the right number of them, as well as their locations^36^. In parallel, methods for identifying a single change point in multiple time series data emerged^37^ and more recently extensions to high-dimensional settings appeared^38^ together with their extension to the case of multiple change points. However, a key assumption has been that the time series under consideration are *independent*, which implies that a simple least squares criterion can be used to identify at least a single change point. On the other hand, the network setting considered in our study obviously violates the latter condition and hence a more complex criterion needs to be employed. Further, as previously mentioned, in the presence of network data streams the objective is not simply to identify transition epochs, but also the nodes in the network that gave rise to them.

Turning our attention to the problem formulation in 2, it can be seen that our interest is in finding the single core common community structure for each of the *M* time epochs, while at the same time detecting and identifying the phase transition time points *τ*_*j*_. Therefore, to achieve this objective, the tuning parameter *λ*(*t*) should be set as large as possible between phase transition time points while at phase transition time points we would like to set *λ*(*t*) as small as possible. Naturally, the second requirement can be achieved by directly removing the total variation penalty, which is equivalent to setting *λ*(*t*) = 0. While for the first requirement, since we assume a fixed low rank component *L*_*m*_ for each stable community time period, we can approximately recover the single low rank component for each period by simply taking the average of the individual low rank components *L*(*t*) recovered without the total variation penalty. Assuming that the length of the time intervals $|{\tau }_{m+1}-{\tau }_{m}|\sim cT,\,c < 1$ scales linearly with time, we would expect that the variations in the estimates of $L(t),t\in ({\tau }_{m},{\tau }_{m+1})$ cancel out and the average low-rank component converges to the true one that generated the weighted network adjacency matrix. Therefore, by considering the following relaxed version of the optimization problem above

3 $$\begin{array}{ll}{\rm{\min }} & \parallel |L(t){\parallel }_{\ast }+\gamma \parallel S(t){\parallel }_{1}+\alpha \parallel E(t){\parallel }_{F}^{2}\\ \,{\rm{s}}.\,{\rm{t}}. & A(t)=L(t)+S(t)+E(t)\end{array}$$

and then averaging the low-rank component estimates between phase transition epochs. Then, the question becomes of how to identify those *τ*_*m*_ epochs accurately. We propose to calculate and monitor over time the *thresholded rank* over time windows of a certain length. This rank is defined as the number of singular values exceeding a carefully selected threshold, which represents the effective number of communities detected. Finally, the core community structure *L*_*m*_ can be estimated by using any standard clustering technique for networks applied to the average ${\overline{L}}_{m}(t)I(t\in ({\tau }_{m},{\tau }_{m+1}))$. A recommended clustering technique for this task is spectral clustering^39,40^ and the overall strategy is illustrated in the next sub-section.

To solve the problem in equation 3, we adopt an alternating splitting augmented Lagrangian method (ASALM) that was proposed in^27^ and is a variant of the widely used alternating direction method of multipliers (ADMM)^41^. The advantage of this method is that its computational complexity of each iteration of the algorithm is dominated by one singular value decomposition (SVD), whose computational efficiency can be further improved by employing a partial SVD decomposition^27,42^ since only leading singular values and corresponding vectors need to be calculated. The augmented Lagrangian function of?? is given by:

4 $$L=\parallel L{\parallel }_{\ast }+\gamma \parallel S{\parallel }_{1}+\alpha \parallel E{\parallel }_{F}^{2}+\langle {\rm{\Lambda }},A-L-S-E\rangle +\frac{\beta }{2}\parallel L+S+E-A{\parallel }_{F}^{2}$$

where *β* > 0 is used to penalize violation of the constraint *A* = *L* + *S* + *E* and $\langle \rangle$ is the trace inner product which is defined as $\langle X,Y\rangle :\,={\sum }_{ij}\,{X}_{ij}{Y}_{ij}$. Based on ASALM, we can minimize the Lagrangian function by splitting it into separate parts and minimize consecutively as follows:

$$\{\begin{array}{rcl}{E}^{k+1} & = & {\rm{argmin}}\,\alpha \parallel E{\parallel }_{F}^{2}+\frac{\beta }{2}\parallel E+{L}^{k}+{S}^{k}-\frac{1}{\beta }{{\rm{\Lambda }}}^{k}-A{\parallel }_{F}^{2}\\ {S}^{k+1} & = & {\rm{argmin}}\,\gamma \parallel S{\parallel }_{1}+\frac{\beta }{2}\parallel S+{L}^{k}+{E}^{k+1}-\frac{1}{\beta }{{\rm{\Lambda }}}^{k}-A{\parallel }_{F}^{2}\\ {L}^{k+1} & = & {\rm{argmin}}\,\parallel L{\parallel }_{\ast }+\frac{\beta }{2}\parallel L+{S}^{k+1}+{E}^{k+1}-\frac{1}{\beta }{{\rm{\Lambda }}}^{k}-A{\parallel }_{F}^{2}\\ {{\rm{\Lambda }}}^{k+1} & = & {{\rm{\Lambda }}}^{k}+\beta ({A}^{k+1}-{L}^{k+1}-{S}^{k+1}-E)\end{array}$$

The Lipschitz constant *β* controls the number of leading singular values we need to compute at each iteration in the general soft singular value thresholding algorithm for low rank matrix recovery/completion, and a lot of related work has been devoted in the area to investigate the strategy of choosing an appropriate value to ensure convergence to the correct results, including dynamically updated ones and fixed ones^27,42,43^. Following^27^, for our purpose we choose a fixed *β* = 0.15*N*^2^/||*A*||_1_, where *N* is the size of the networks.

Therefore, incorporating results from^43,44^, we have the following iterative updating algorithm for iteration *q*
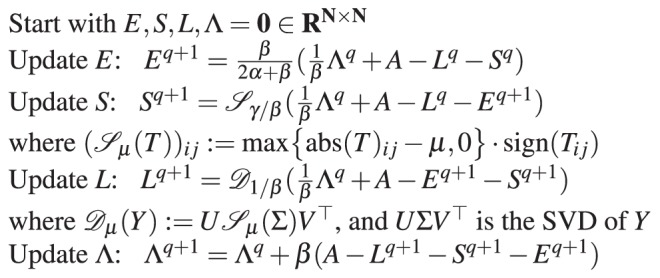


Note that the update for the dense noise component *E* involves a closed form formula, that for the sparse component a soft-thresholding step, while that for the low-rank component a SVD. Finally, the update of the Lagrange multipliers Λ is given by another closed form formula. The stopping condition for the algorithm is given by: $\frac{\parallel {L}^{q+1}-{L}^{q}{\parallel }_{F}^{2}+\parallel {S}^{q+1}-{S}^{q}{\parallel }_{F}^{2}}{\parallel {L}^{q}{\parallel }_{F}^{2}+\parallel {S}^{q}{\parallel }_{F}^{2}+1}\le \varepsilon$.

To employ the above algorithm, the tuning parameters *α* and *γ* need to be specified. Further, once the network adjacency matrices have been decomposed, the task becomes on how to identify the phase transition epochs and determine the number of communities and their membership. These issues are discussed next and illustrated on synthetic data generated according to the following mechanism.

#### Data Generation for Illustrative Example

**Figure 1:**
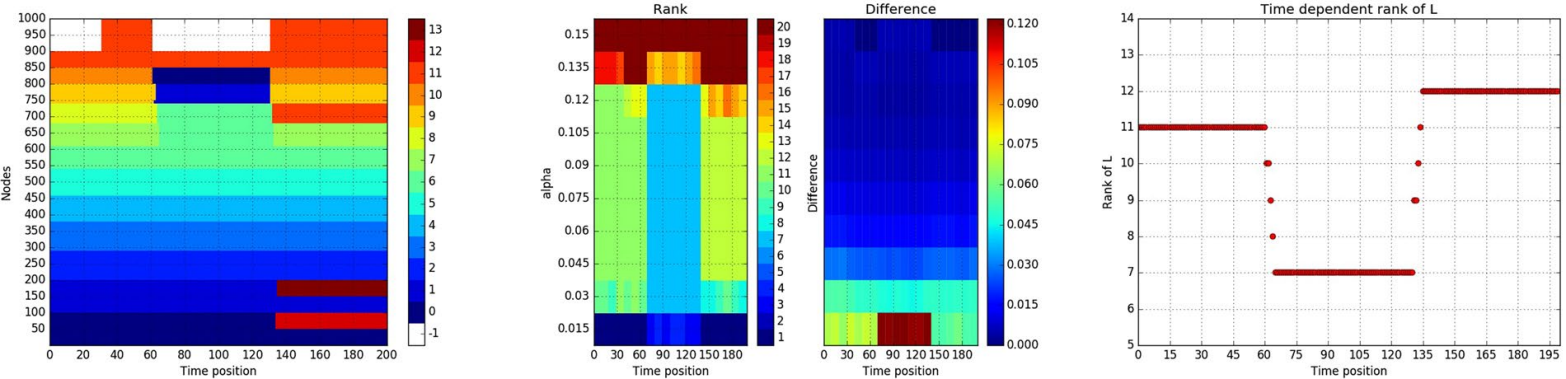
(**a**) Left panel, design of the time dependent community structure. (**b**) Middle panel, illustration of rank and inconsistency across time based on a range of values of *α*. (**c**) Right panel, rank of *L*(*t*) recovered based on *α* = 0.1

We employ a statistical factor model to generate the sequence of network adjacency matrices. For each of the *M* stable time periods, a common low rank component $L(t)={L}_{m}={U}_{m}{U}_{m}^{T}$ is generated for all $t\in ({\tau }_{m},{\tau }_{m+1})$, where each column *u*_*k*_(*m*) of *U*_*m*_ satisfies that for nodes belonging to community *k*, ${u}_{k}{(m)}_{i}\mathop{\sim }\limits^{i.i.d.}Unif({r}_{in},1)$ and for those nodes not in community *k*, ${u}_{k}{(m)}_{i}\mathop{\sim }\limits^{i.i.d.}Unif(0,{r}_{out})$. It can be seen that by selecting parameters so that *r*_*in*_ > *r*_*out*_, a node *i* belongs to community *k* if *u*_*ik*_ ≥ *r*_*in*_. Finally, the sparse and dense noise components, at each time point *t* are generated according to:

- $$S(t)={S}_{1}(t)\,\bullet \,{S}_{2}(t)$$, where $${S}_{1}(t)\mathop{\sim }\limits^{i.i.d.}Bernoulli({p}_{s})$$, $${S}_{2}(t)\mathop{\sim }\limits^{i.i.d.}Unif(\,-\,1,1)$$.
- $$E(t)\mathop{\sim }\limits^{i.i.d.}Unif(\,-\,r,+r)$$, *r* > 0.
- $$A(t)={{\mathscr{P}}}_{{\rm{\Omega }}}(L(t)+S(t)+E(t))$$, Euclidean projection onto the space $${\rm{\Omega }}:=\{X\in {{\bf{R}}}^{N\times N}|\mathrm{\ 0}\le {X}_{i,j}\le 1,$$$${X}_{ij}={X}_{ji}\}$$

where $X\,\bullet \,Y=[{X}_{ij}{Y}_{ij}]$ for $X,\,Y\in {{\bf{R}}}^{{\bf{M}}\times {\bf{N}}}$ denotes element wise matrix multiplication, *p*_*s*_ is the density of the sparse noise, and *r* is the upper bound for the magnitude of the dense noise component. For our illustrative example, we set the following values for the control parameters for the model: *r*_*out*_ = 0.4, *r*_*in*_ = 0.6, *p*_*s*_ = 0.1 and *r* = 0.1, and we generate *T* = 200 network snapshots, divided into *M* = 4 stable periods, with 11, 11, 7, and 12 densely connected communities respectively, and phase transition epochs occurring at times *τ* = 30–31, 60–65, 130–135. The time dependent community membership is depicted in panel (a) of Fig. 1 with community *-1* representing the missing nodes and the detail of the network sequence is as follows: At *t* = 0, we set the network size as *N* = 900 and there are 11 densely connected communities numbered from 0 to 10 of size 100, 100, 90, 90, 80, 80, 70, 70, 60, 60, 50, respectively, with the remaining 50 nodes not belonging to any community and hence expected to be captured by the noise component. For pure notation purposes, we call these 50 outlying nodes, the “11th community.” At time 31, an extra 100 nodes not exhibiting any community structure join the network and hence are assigned to the “11th community.” From time 61–65 the 100 nodes that joined the network at time 31 leave, while community 10 joins 0, 9 joins 1, and 7 and 8 join 6. From times, 131–135, the network structure reverts to the one being present between time periods 31–60, with the addition that community 8 disintegrates and joins the “11th community”, and communities 1 and 2 split into two separate communities each. This structure persists till the the end. It can be seen that a number of intricate changes occur to the network structure designed to showcase the power of the proposed methodology.

#### Selection of tuning parameters and decomposition

There are two parameters required in the optimization problem above; following suggestions in literature, we recommend selecting $\gamma =1/\sqrt{N}$ for general purpose^45^. For *α*, extensive numerical work shows that searching over the range $(0.5/\sqrt{N+\sqrt{8N}},10/\sqrt{N+\sqrt{8N}})$ provides highly satisfactory results.

For our problem we sample 20 evenly spaced time points from the total *T* = 200 and plot the rank of the recovered low rank components for these time points across different values of *α*. To facilitate identifying the optimal *α* we also include the plot of inconsistency/difference of the *L*(*t*) across consecutive values of *α*, defined as $\parallel L{(t)}_{{\alpha }_{i}}-L{(t)}_{{\alpha }_{i+1}}{\parallel }_{F}/\parallel L{(t)}_{{\alpha }_{i}}+1{\parallel }_{F}$ with *α*_*i*+1_ − *α*_*i*_ = 0.015 in our case. The results are shown in Fig. 1(b), and in order to recover robust *L*(*t*) components, we suggest choosing a value of *α* that would result in low inconsistency from the wide range of *α* values that would recover consistent rank across time points. For example, in our case, the wide range is $\alpha \in [0.045,\,0.105]$ and we choose *α* = 0.1 to minimize the inconsistency.

With the above optimal set of parameters, the rank of the recovered *L*(*t*) across time is shown in Fig. 1(c) and simply by inspecting the evolution of rank across time, we can easily identify the two groups of phase transition time points around *t* = 60 and *t* = 130. However, this is not a very reliable method because on the one hand we did not capture the phase transition time points around *t* = 30 and on the other hand in terms of real life applications the rank of *L*(*t*) might not be so clean. Therefore, we develop the following strategy based on the *thresholded rank* of averaged *L*(*t*) to accurately and robustly identify phase transition time points.

#### Phase transition identification

**Figure 2:**
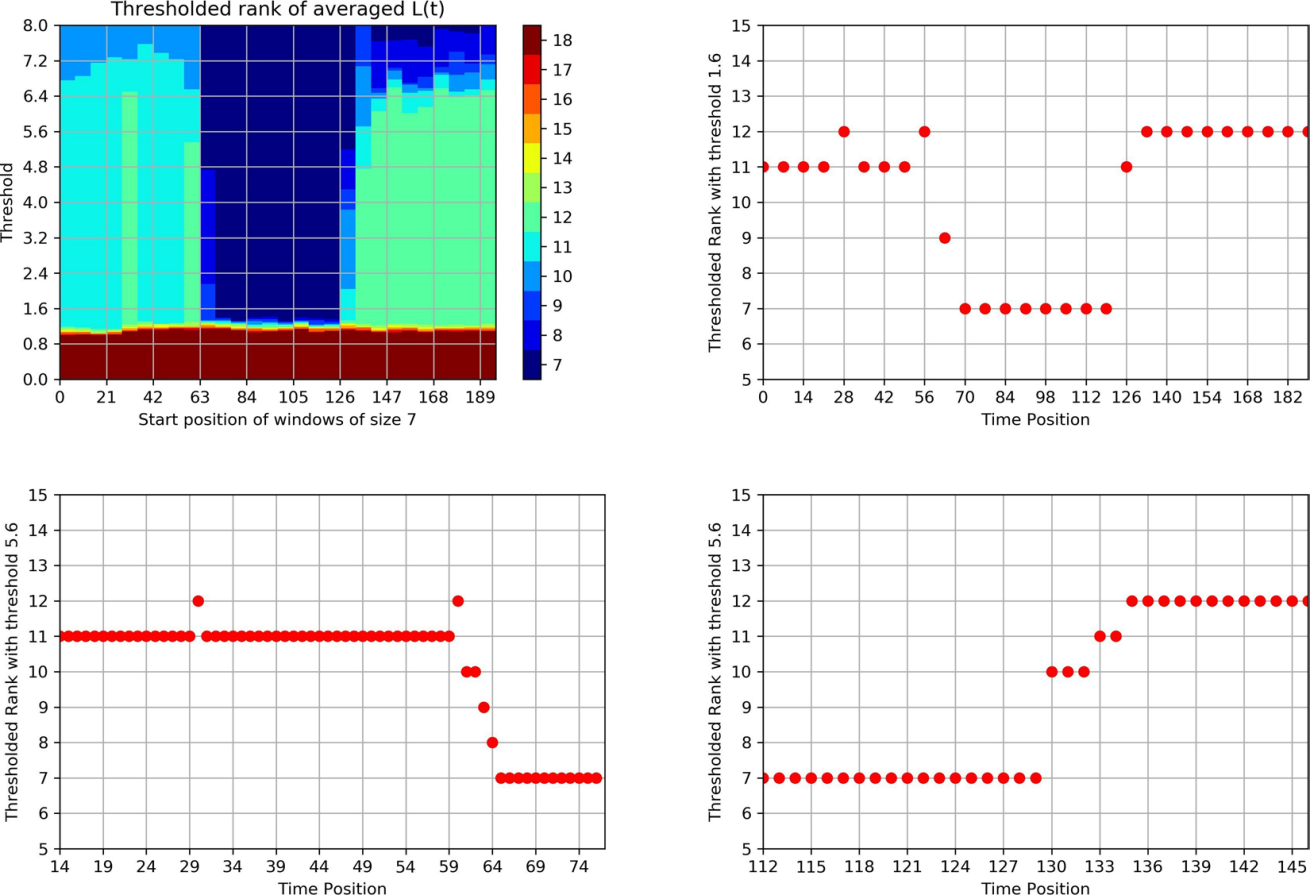
(**a**) Upper left panel, *thresholded rank* of windows of length 7 across time, based on different values of the *threshold*. (**b**) Upper right panel, *thresholded rank* of windows of length 7. (**c**) Lower left panel, *thresholded rank* by zooming-in using windows of length 2 around the first two phase transition epochs. (**d**) Lower right panel, *thresholded rank* of zoom-in windows of length 2 around the last phase transition epochs.

The strategy of identifying phase transition epochs {*τ*_*m*_} is as follows. First, split the *T* available time points into $\sqrt{T}$ non-overlapping windows of equal length $\sqrt{T}$. Then, scan over these windows and within each window compute for a range of values of thresholds the *thresholded rank* of the averaged low rank component, defined as the number of singular values exceeding the threshold. If a window is within a certain stable period, following the argument in the section of model formulation, we would expect an enhanced modularity and therefore the *thresholded rank* would be consistent with the number of communities in the network across a wide range of threshold values. Therefore, if on one hand, the length of stable periods exceeds the window size, then we would expect a consistent *thresholded rank* across windows. On the other hand, if the window covers time points from two different stable periods having different community structures, the *thresholded rank* will exhibit volatility until it belongs to the new stable time period, wherein the *thresholded rank* starts to exhibit a stable behavior. Thus, to find the phase transition epochs, we simply choose an appropriate threshold value from a wide range of such values that would result in consistent *thresholded rank* across windows and identify the windows where the thresholded *rank* first exhibits volatility. For illustration purposes, we choose a window of length 7 and the wide range of threshold values is approximately $h\in [1,\,6]$ and we choose *h* = 1.6 for our purpose as is shown in (a) and (b) of Fig. 2. The choice of the window length is supported by some theoretical work^46^ and by extensive numerical experimentation. Of course, the accuracy level of the identification is limited by the window length ~$\sqrt{T}$ and to improve accuracy, around the phase transition time points obtained from above, we can zoom in to windows of smaller length, say ~$\sqrt{\sqrt{T}}$, and repeat the same procedure. Naturally, the window length can be further reduced to obtain a desired level of resolution for identifying the phase transition epochs. The selection of a $\sqrt{T}$ window length is dictated by the need for scalability of the procedure, in the presence of data sets involving a very large number of time points *T*.

To illustrate the idea of the window scanning strategy, we consider the results of our illustrative example as shown in Fig. 2. From the first level of windows of length 7 we identify the phase transition positions around *t* = 28, 56~63, 126. Then we zoom in around these time points with window length 2 and identify the phase transition time points *t* = 31, 61~65, 131~135 as shown in (c) and (d) of Fig. 2. Here in order to scan windows over the whole time series, we cast the adjacency matrices of all networks with smaller size to matrices of size *N*_*max*_, the maximum size of networks in the whole time range, and set entries 0 for those missing nodes. Note here we identified the phase transition time position *t* = 30 when new nodes join the network, while if we simply look at the time dependent rank of *L*(*t*) in Fig. 1(c) we would fail to detect this since the two regimes share the same rank but different community structures.

#### Core community structure detection

The final step involves identifying the invariant core community structures between phase transition epochs based on averaged ${\overline{L}}_{m}(t)$. In our example, the average misclassification rate is 0.24% when clustering based on each individual *L*(*t*) and 0% based on averaged ${\overline{L}}_{m}(t)$. For comparison, if we cluster simply based on the original time series adjacency matrices *A*(*t*) corrupted with both dense and sparse noises, the average misclassification rate is as high as 0.89%.

### Application to Synthetic Network Data

We employ the following three mechanisms to induce community structure in the sequences of networks snapshots observed. (i) a factor model, (ii) a stochastic block model (SBM) and (iii) a weighted stochastic block model (WSBM)^15,47^. For simplicity, networks generated by the three models share the same time patterns on when the community structure changes. There are *T* = 30 network snapshots in total, divided in *M* = 3 stable time periods and all networks have size *N* = 1000 nodes. At *t* = 0, there are 11 densely connected communities numbered from $0,\ldots ,10$ of size 120, 100, 100, 100, 80, 80, 70, 70, 70, 60, 60, while the remaining 90 nodes do not form any community. At time *t* = 10 community 10 joins 0 and 9 joins 1 and this structure is fixed till *t* = 19. At time *t* = 20, community 7 and 8 join together with 6 and similarly this structure is fixed till the end.

The specifics of the network formation mechanisms are described next:

(i) Factor model: We fix *r*_*out*_ = 0.4, *r*_*in*_ = 0.6 for each *L*(*t*) and control the density *p*_*s*_ of the sparse noise and the magnitude *r* of the dense noise, which are generated the same way as described in the illustrative example.
(ii) SBM: Only sparse noise is introduced in this case since the entries of the adjacency matrices are binary. We fix the density of noise to be 0.1 and control the density of connections *p*_*c*_ within communities for each *L*(*t*).

- Generate ${L}_{in}\mathop{\sim }\limits^{i.i.d.}Bernoulli\,({p}_{c})$, and *L*_*out*_ = 0, where *L*_*in*_ stands for connections within communities and *L*_*out*_ for otherwise.
- Generate $S\mathop{\sim }\limits^{i.i.d.}Categorical\,(\pi )$, such that *P*(*S*_*ij*_ = 1) = 0.05, *P*(*S*_*ij*_ = −1) = 0.05 and *P*(*S*_*ij*_ = 0) = 0.9.
- Project to adjacency matrices: $A(t)={{\mathscr{P}}}_{{\rm{\Omega }}}[L(t)+S(t)]$.

(iii) WSBM: Similar to the factor model, we fix *L*(*t*) by generating them in the following way: ${L}_{in}\mathop{\sim }\limits^{i.i.d.}Unif(0.4,1)$ and ${L}_{out}\mathop{\sim }\limits^{i.i.d.}Unif(0,0.6)$. Then, the strength of the sparse and dense noise is generated in the same way as before. Note if we have an adequate number of network snapshots, the connection strength of ${\overline{L}}_{m}(t)\to \frac{1+0.4}{2}=0.7$ for edges belonging to communities and is larger than ${\overline{L}}_{m}(t)\to \frac{0+0.6}{2}=0.3$ for edges connecting different communities, although for any individual low-rank component *L*(*t*) the connection strength of some edges might not be concordant with this ranking.

For both the factor and WSBM models, we design dense and sparse noise components with various levels of strength controlled by *r* or *p*_*s*_ as shown in Tables 1 and 2, while for the SBM model we control *p*_*c*_ to study the performance having different connection strength within communities, as given in Table 3. Applying the proposed methodology, we first successfully identify the phase transition time points *t* = 9, 19 for all the cases with optimal values for parameters *γ*,*α* given in the respective Tables. Subsequently, we compare the performance of community detection based on individual low-rank components *L*(*t*), averaged ones ${\overline{L}}_{m}$ over the stable periods and the original adjacency matrices *A*(*t*). For the factor model, the misclassification rate based on the recovered *L*(*t*) from *A*(*t*) are consistently lower than *A*(*t*), while for the SBM and WSBM models, this is not necessarily the case. However, for all the scenarios examined, the misclassification rate based on the averaged ${\overline{L}}_{m}$ goes to zero except for some extreme cases. Note that the *recovery rate* is defined as the proportion of densely connected communities recovered, while the *error rate* is the averaged misclassification rate across time for those recovered communities.

**Table 1:** Dense noise case for factor model and WSBM.

Models	r	*γ*	*α* (×10^−2^)			Recovery Rate			Error Rate (×10^−3^)								
			t1	t2	t3	t1	t2	t3	t1			t2			t3		
									*A*(*t*)	*L*(*t*)	$\overline{{\boldsymbol{L}}}$	*A*(*t*)	*L*(*t*)	$\overline{{\boldsymbol{L}}}$	*A*(*t*)	*L*(*t*)	$\overline{{\boldsymbol{L}}}$
Factor	0.3	0.03	4.5	4.5	4.5	1	1	1	48	11	0	48	6	0	37	3	0
	0.4	0.03	3.2	2.8	2.8	1	1	1	61	24	0	41	12	0	33	6	0
	0.5	0.02	3.9	3.5	3.5	1	1	1	103	192	3	63	78	0	42	36	0
WSBM	0.5	0.02	3.5	3	3	1	1	1	0	1	0	0	0	0	0	0	0
	0.7	0.02	3	2.8	2.8	1	1	1	10	41	0	4	12	0	2	2	0
	0.9	0.02	2.1	2.1	2.1	4/11	4/9	5/7	76	223	0	78	130	0	46	57	0

**Table 2:** Sparse noise case for factor model and WSBM.

Models	*p*_*s*_	*γ*	*α* (×10^−2^)			Recovery Rate			Error Rate (×10^−3^)								
			t1	t2	t3	t1	t2	t3	t1			t2			t3		
									*A*(*t*)	*L*(*t*)	$\overline{{\boldsymbol{L}}}$	*A*(*t*)	*L*(*t*)	$\overline{{\boldsymbol{L}}}$	*A*(*t*)	*L*(*t*)	$\overline{{\boldsymbol{L}}}$
Factor	0.5	0.02	15	4.5	4.5	1	1	1	183	3	0	129	18	0	81	11	0
	0.6	0.02	6	4.3	4.3	1	1	1	344	40	0	171	35	0	104	22	0
	0.7	0.02	4.5	4	4	1	1	1	515	169	36	273	109	0	150	47	0
WSBM	0.5	0.02	3.5	3	3	1	1	1	1	1	0	1	1	0	0	0	0
	0.7	0.02	3.4	3.2	3	1	1	1	19	32	0	8	10	0	2	2	0
	0.9	0.02	2.1	2.1	2.1	4/11	4/9	5/7	68	174	0	65	102	0	76	55	0

**Table 3:** Sparse noise case for SBM.

Models	*p*_*c*_	*α*	*γ* (×10^−2^)			Recovery Rate			Error Rate (×10^−3^)								
			t1	t2	t3	t1	t2	t3	t1			t2			t3		
									*A*(*t*)	*L*(*t*)	$\overline{{\boldsymbol{L}}}$	*A*(*t*)	*L*(*t*)	$\overline{{\boldsymbol{L}}}$	*A*(*t*)	*L*(*t*)	$\overline{{\boldsymbol{L}}}$
SBM	0.3	0.3	5.5	5	4.5	1	1	1	5	9	0	3	6	0	2	3	0
	0.2	0.2	6.1	5.7	5.3	1	1	1	51	59	0	43	57	0	14	18	0
	0.1	0.3	20	20	20	1	1	1	556	466	7	366	459	58	281	377	10

**Figure 3:**
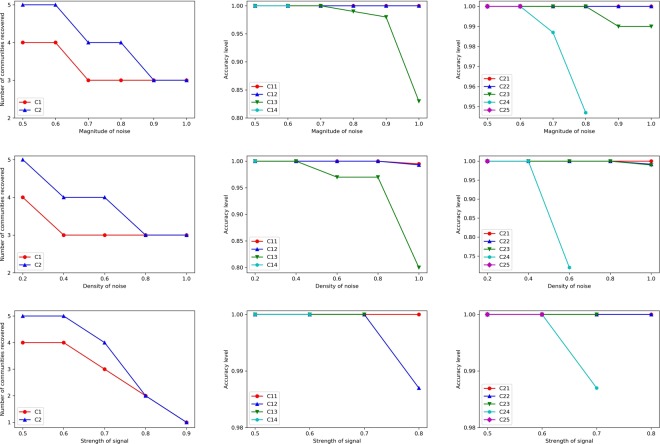
Upper row (**a**–**c**) presence of only dense noise, with *ub* = 0.7. Middle row (**d**–**f**) presence of both dense noise (*r* = 0.5) and varying degree of density of sparse noise with *ub* = 0.7. Lower row (**g**–**i**) fixed dense and sparse noise (*r* = 0.5, *p*_*s*_ = 0.5), and varying signal strength (*ub*). (a) Upper left panel, number of communities detected versus magnitude of dense noise *r*. (b) Upper middle panel, accuracy level of recovery for each community during the first stable period, with *C*11 representing the community of size 200, *C*12 that with 150 nodes, *C*13 with 100 and *C*14 with 50 nodes. (c) Upper right panel, accuracy level of recovery for each community during the second stable period, with *C*21 representing the community of size 150, *C*22 of 125, *C*23 of 100, *C*24 of 75 and *C*25 of 50 nodes, respectively. (d) Middle left panel, number of communities detected versus density of sparse noise *p*_*s*_. (e) Middle middle panel, accuracy level of recovery for each community during the first stable period. (f) Middle right panel, accuracy level of recovery for each community during the second stable period. (g) Lower left panel, number of communities detected versus signal strength *ub*. (h) Lower middle panel, accuracy level of recovery for each community during the first stable period. (i) Lower right panel, accuracy level of recovery for each community during the second stable period.

To further demonstrate the effectiveness of the proposed methodology in both identifying phase transition epochs and in accurately recovering core community structures, we design the following time evolving networks of size *N* = 500 based on the WSBM model. There are *T* = 40 network snapshots in total, with $t=1,\ldots ,20$ sharing the same community structure with respective sizes 200, 150, 100, and 50. At time *t* = 21 community 1 of size 200 splits into 2 of size 125 and 75 respectively, and this structure persists until *T* = 40. For obtaining the adjacency matrices, we set ${L}_{out}\mathop{\sim }\limits^{i.i.d.}{\rm{Unif}}(0,ub)$ and ${L}_{in}\mathop{\sim }\limits^{i.i.d.}{\rm{Unif}}(1-ub,1)$ and control the *signal strength* by varying *ub*. Further, we incorporate various levels of dense and sparse noise by controlling *r* and *p*_*s*_. As is shown in Fig. 3, when the noise level increases or equivalently the signal strength decreases, small communities tend to be captured by either the sparse or dense noise components, and only communities of larger size can be recovered. However, as long as a community is detected, its members can be recovered with very high accuracy across various levels of noise. As for phase transition epochs detection and identification, since we monitor the *thresholded rank* of averaged low rank matrices, as long as the change is driven by changes in the communities recovered by the proposed procedure, they can be also successfully identified. Indeed, this is the case for all the scenarios tested, except when *ub* ≥ 0.9; the latter case is depicted in Fig. 3(g) where no core community can be detected when *ub* ≥ 0.9 and thus the proposed phase transition detection procedure fails. This demonstrates the overall robustness of the proposed procedure, even in the presence of fairly high noise in the data.

In summary, across three network formation models and a wide range of scenarios examined, the proposed methodology is capable of correctly identifying phase transition epochs, accurately estimate the number of communities, as well as their membership.

### Time-Evolving Synchronization Patterns in the Kuramoto Model

Next, we investigate synchronization patterns on networks over time by the dynamical system of Kuramoto oscillators. This model has been extensively studied in the literature from various angles. In this work, we construct and study the behavior of resulting networks in the following two settings, also examined in^19,48^: time evloving community structures and two level hierarchical community structures. In both settings, there are *N* = 256 coupled phase oscillators in total and the phase *θ*_*i*_(*t*) of the *i*-th oscillator evolves in time according to

5 $$\frac{d{\theta }_{i}}{dt}={\omega }_{i}+\sum _{j}\,\kappa {C}_{ij}\,\sin ({\theta }_{j}-{\theta }_{i})\,i=1,\ldots ,N$$

where ${\omega }_{i}\mathop{\sim }\limits^{i.i.d.}Normal\,(0,\,1)$ denotes the natural (initial) phase of the oscillators, *κ* = 0.25 is the coupling strength, and *C* is the support coupling matrix, such that oscillators *i* and *j* are coupled, if and only if *C*_*ij*_ = 1. Mapping each oscillator to a node of a network, then the evolution of the model resorts to a time evolving network with the strength of connections represented by the similarities

6 $${A}_{ij}(t)=\langle |\,\cos \,[{\theta }_{i}(t)-{\theta }_{j}(t)]|\rangle $$

where the angular brackets stands for the average over 40 different initial random phases.

**Figure 4:**
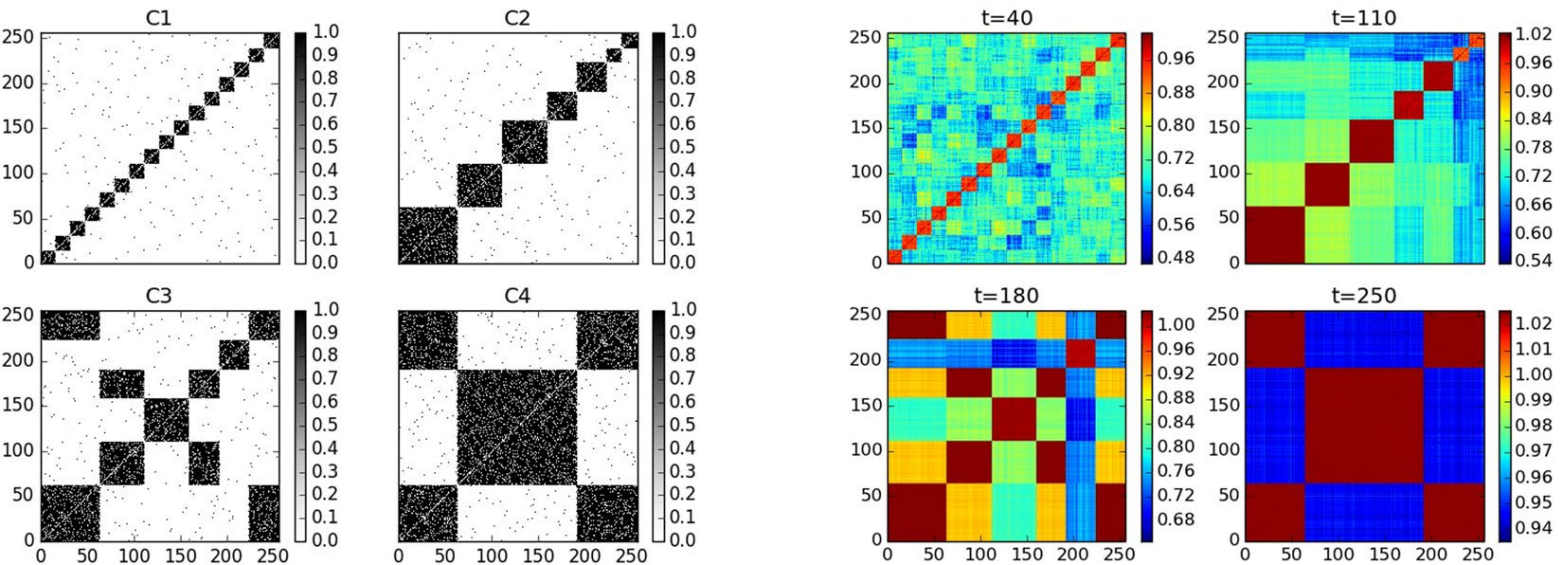
(**a**) Left panel, support coupling matrices for the 4 stable periods. (**b**) Right panel, adjacency matrices at times 40, 110, 180 and 250.

In the first experimental setting, the total number of network snapshots is *T* = 280, with phase transition epochs occuring at times *τ*_1_ = 70, *τ*_2_ = 140 and *τ*_3_ = 210. To illustrate the structure of the resulting networks, the support coupling matrices and the corresponding adjacency matrices at times 40, 110, 180 and 250 are depicted in (a) and (b) of Fig. 4. Further, the structure of the support matrices where the communities correspond to the dark-colored nodes is generated as follows: for each node in community *k* of size *N*_*k*_, there are exactly 14*N*_*k*_/16 connections with nodes inside the community, and 1 with nodes outside of it. In stable period 1 (i.e. for all *t*≤*τ*_1_), there are 16 equal size communities comprising of *N*_*k*_ = 16 nodes each, indexed by $0,\ldots ,15$; in stable period 2 ($t\in ({\tau }_{1},{\tau }_{2})$) communities 1, 2 and 3 merge with community 0, 5 and 6 with 4, 8 and 9 with 7, 11 with 10, and 13 with 12; in stable period 3 ($t\in ({\tau }_{2},{\tau }_{3})$), communities 14 and 15 merge with the enlarged community 0, community10 with 4 and finally in the last stable period (*t* ≥ *τ*_3_) community 12 merges with 0, and 7 with 4. Time steps for synchronization for the 4 periods are *τ* = 0.1, 0.08, 0.05 and 0.02 respectively.

**Figure 5:**
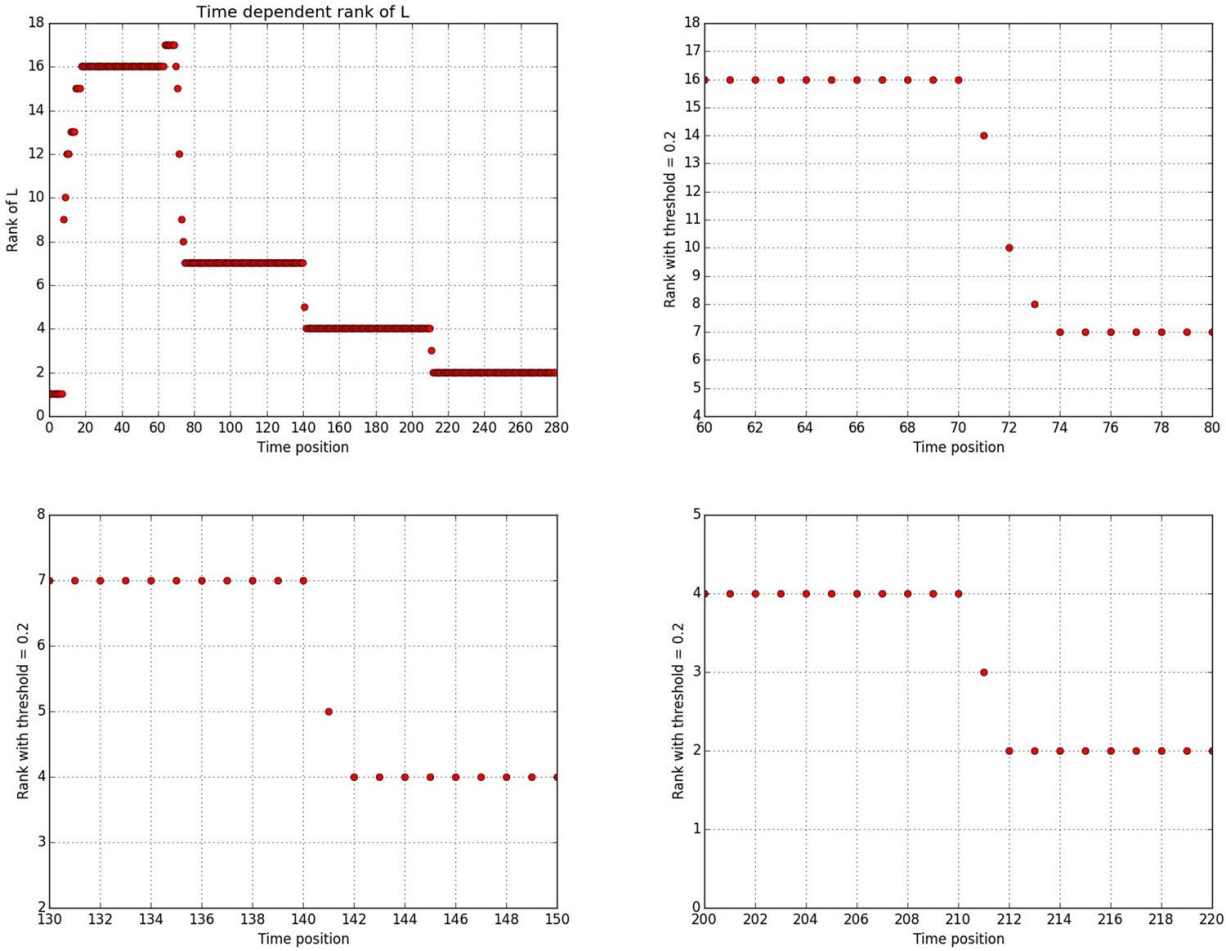
(**a**) Upper left panel, time dependent rank of *L*(*t*) recovered. (**b**) Upper right panel, *thresholded rank* of windows of length 2 around the first phase transition epoch. (**c**) Lower left panel, *thresholded rank* of windows of length 2 around the second phase transition epoch. (**d**) Lower right panel, *thresholded rank* of windows of length 2 around the third phase transition epoch.

**Figure 6:**
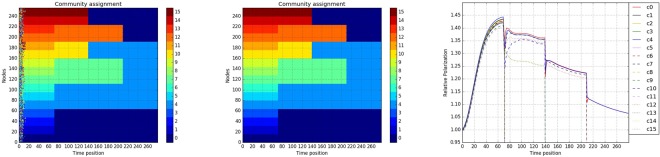
(**a**) Left panel, community membership based on each individual *L*(*t*). (**b**) Middle panel, community membership based on averaged ${\overline{L}}_{m}$ of the 4 stable periods. (**c**) Right panel, relative polarization of communities based on ${\overline{L}}_{m}$ across time.

Following the strategy previously outlined for the selection of tuning parameters in the posited optimization problem, we identify the optimal *α* = 0.72, based on which the rank of recovered *L*(*t*) is depicted in Fig. 5(a). With the final zoomed-in windows of length 2 shown in Fig. 5(b–d) we estimate the 3 phase transition epochs as follows: ${\tau }_{1}\in (71,73),$ *τ*_2_ = 141 and *τ*_3_ = 211. Note that in the presence of several small communities merging from stable period 1 to 2, there is some variability in the estimate of the epoch, while the other two epochs are identified precisely. Finally, we calculate the average of the low-rank matrices *L*(*t*) in these four stable periods and cluster them to extract their community structure. The community membership extracted from all 280 snapshots (by clustering the *L*(*t*) matrices) and by the average ones over stable periods are shown in (a) and (b) of Fig. 6. It can be seen that the average ones provide a much clearer identification of the members in each community.

To further investigate the cohesion of communities at different points in time, we also introduce a new metric coined *relative polarization* for a certain community *k*

7 $${P}_{k}(t)=\frac{{\sum }_{i,j=1}^{{N}_{k}(t)}\,{A}_{ij}^{(k)}(t)}{{N}_{k}^{2}(t)\times {P}_{out}(t)},\,{\rm{with}}\,{P}_{out}(t)=\frac{{\sum }_{i,j=1}^{N}\,{A}_{ij}(t)-{\sum }_{i,j=1}^{{N}_{k}(t)}\,{A}_{ij}^{(k)}(t)}{{N}^{2}-{\sum }_{k=0}^{K-1}\,{N}_{k}^{2}(t)}$$

where *N*_*k*_(*t*) is the size of community $k\in \{0,\ldots ,K-1\}$ at time *t* and ${A}_{ij}^{(k)}(t)$ denotes the edge weight between nodes *i* and *j* in community *k* at time *t*. Intuitively speaking, the relative polarization of community *k* is the ratio between the average connection strength within community *k* and the average strength between all communities.

The relative polarization of each community consistently increased during the first stable time period, while in the following stable time periods this pattern reversed, which indicates that the intracommunity synchronization process within each community is dominant in early stages, while in the later stable periods the inter-community synchronization process becomes more important.

In the second experimental setting, we investigate the synchronization process of the Kuramoto model generated network, exhibiting hierarchical structure. Specifically, each network comprises of 256 nodes (oscillators) evolving across *T* = 100 time points. The coupling strength is *κ* = 0.25, time step for synchronization over the whole time range is *τ* = 0.1, and the adjacency matrices are obtained based on an average over 40 different initial random phases. In the whole range, the community structure is fixed, and the support coupling matrices are designed as follows: there are 16 equally sized first level communities, and 4 equally sized second level communities. For each node, there are exactly 15 connections with nodes in the first level community, 3 in second level (outside the first level) and 1 with nodes outside both levels. This hierarchical structure corresponds to 16 tightly coupled communities that are also organized in 4 more loosely coupled ones. Hence, the presence of this hierarchical structure makes the identification of phase transition epochs, as well as the community structures a challenging problem.

**Figure 7:**
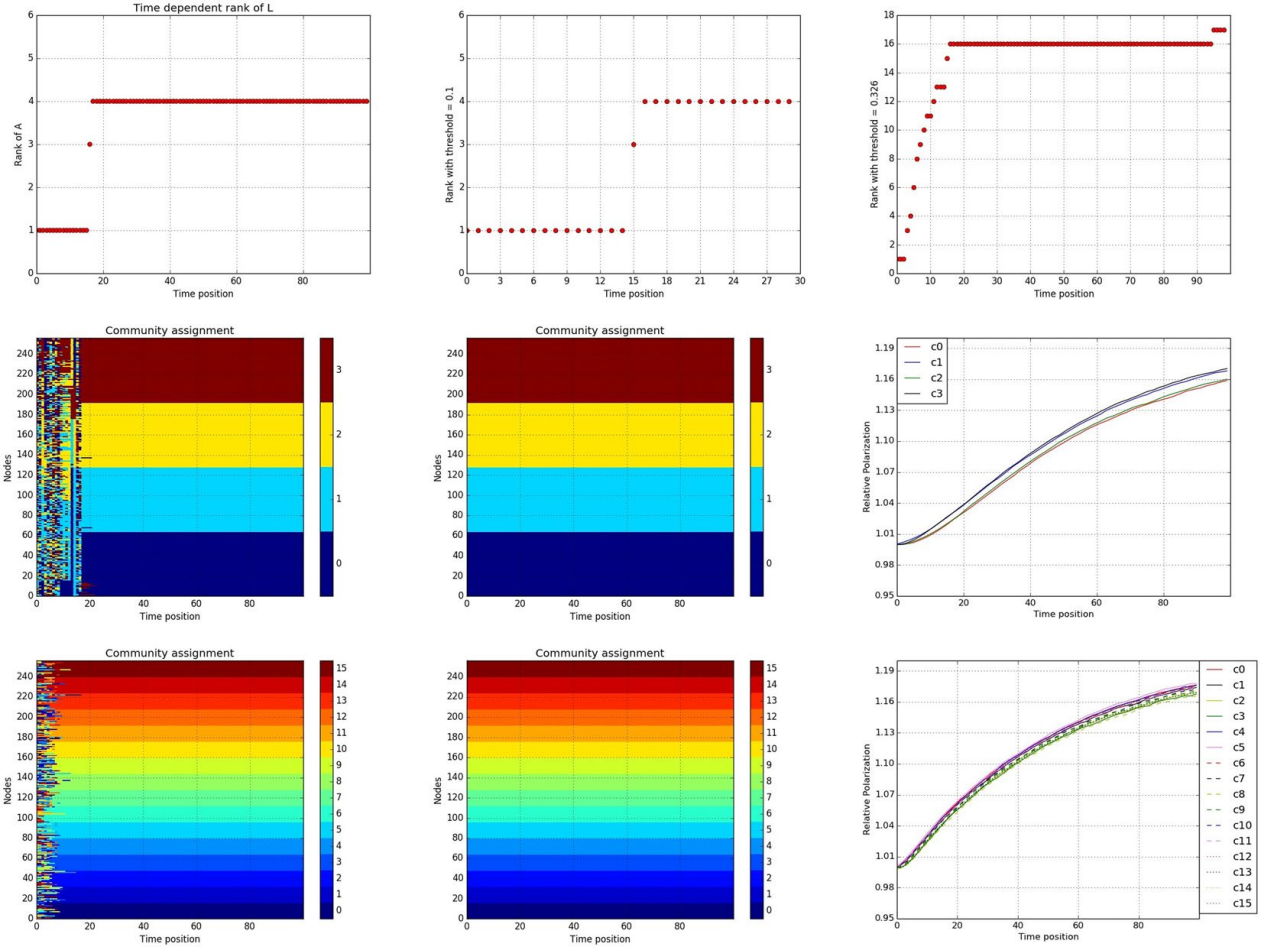
(**a**) Upper left panel, rank of recovered *L*(*t*) across time. (**b**) Upper middle panel, *thresholded rank* of windows of length 2 around the first possible *phase transition epoch* for *L*(*t*). (**c**) Upper right panel, *thresholded rank* of windows of size 2 over the whole time range for *E*(*t*). (**d**) Middle left panel, community membership based on individual *L*(*t*). (**e**) Middle middle panel, community membership based on averaged $\overline{L}$ for *t* ≥ 16. (**f**) Middle right panel, relative polarization of communities based on $\overline{L}$ over time. (**g**) Lower left panel, community membership based on individual *E*(*t*). (**h**) Lower middle panel, community membership based on averaged $\overline{E}$ for *t* ≥ 16. (**i**) Lower right panel, relative polarization of communities based on $\overline{E}$ over time.

First, we find the optimal *α* = 0.35, based on which the the rank of recovered *L*(*t*) is depicted in Fig. 7(a). What is interesting is that the dense noise component *E*(*t*) actually captures the first level community structure, as can be seen from the results base on window scanning of length 2 in Fig. 7(c). A first sight on 7(b), the *thresholded rank* of averaged *L*(*t*) of window length 2 might indicate a phase transition around *t* = 16, but a closer look will show that there is no community structure in the first *stable* period since the *thresholded rank* is 1. Therefore, we try to recover community structure based on the *thresholded rank* at time *t* ≥ 16 for both *L*(*t*) and *E*(*t*) in the whole time range. Not surprisingly, based on (d) and (g) of Fig. 7 neither the first level nor the second level community structure forms concretely until approximately time *t* = 20 when the community memberships based on *L*(*t*) and *E*(*t*) start to match those based on $\overline{L}$ and $\overline{E}$.

### Application to U.S. Senate Roll Call Voting Data

A topic of great interest in the political science literature is that of *polarization*, as well as understanding formation of coalitions (groups) over time of members of legislative bodies. Selected prior work includes the work in^49^ that analyzed roll call data of the US Congress from 1879 to 1987 (both for the House of Representatives and the Senate), defined a distance measure between the two political parties and calculated its evolution over the corresponding 90-year period. More recently^17,50^, examined roll call data from the US Senate for the period 1979 to 2012 and used a community modularity quality function to study the issue of polarization. Their key finding is that modularity exhibits a sharp change around early 1995, with members of the two parties drifting apart in their voting pattern. In an attempt to go beyond exploratory analysis^21^, developed a formal estimation framework for the presence of a single change point (phase transition) based on probabilistic graphical models and confirmed the main finding in^17,50^. Other related findings regarding the time evolving community structures of legislative bodies can be found in^51^ based on US Senate roll call data and using synchronization methods, and in^52^ that examined legislation bill co-sponsorship networks of the Peruvian Congress and successfully captured the power shifts during the 2006–2011 period using time dependent community detection based on multilayer modularity maximization.

Before discussing the results, we briefly present the necessary data preprocessing steps undertaken. We examine all roll call votes of the US Senate from the 96th to the 113th Congress, covering the period 1979–2014. The nodes of the network represent a specific Senate seat for each state and we mapped the voting records of individual Senators over time to the corresponding seats, ensuring continuity of the voting record for each seat. For each Congress, the adjacency matrix is constructed element-wise as follows: ${A}_{ij}=\frac{1}{{S}_{ij}}{\sum }_{k=1}^{K}\,{c}_{ij}^{(k)}$, where *S*_*ij*_ is the total number of votes that both senators *i* and *j* participated in, *K* is the total number of votes that particular Congress undertook and ${c}_{ij}^{(k)}=1$ if they voted the same way and 0, otherwise. The reason we consider the time-evolving roll call voting record network at the Congress time-scale is that election times that occur every two years wherein 1/3 of the Senate members are up for election represent the main external events that can induce changes in the network composition, including its community structure.

**Figure 8:**
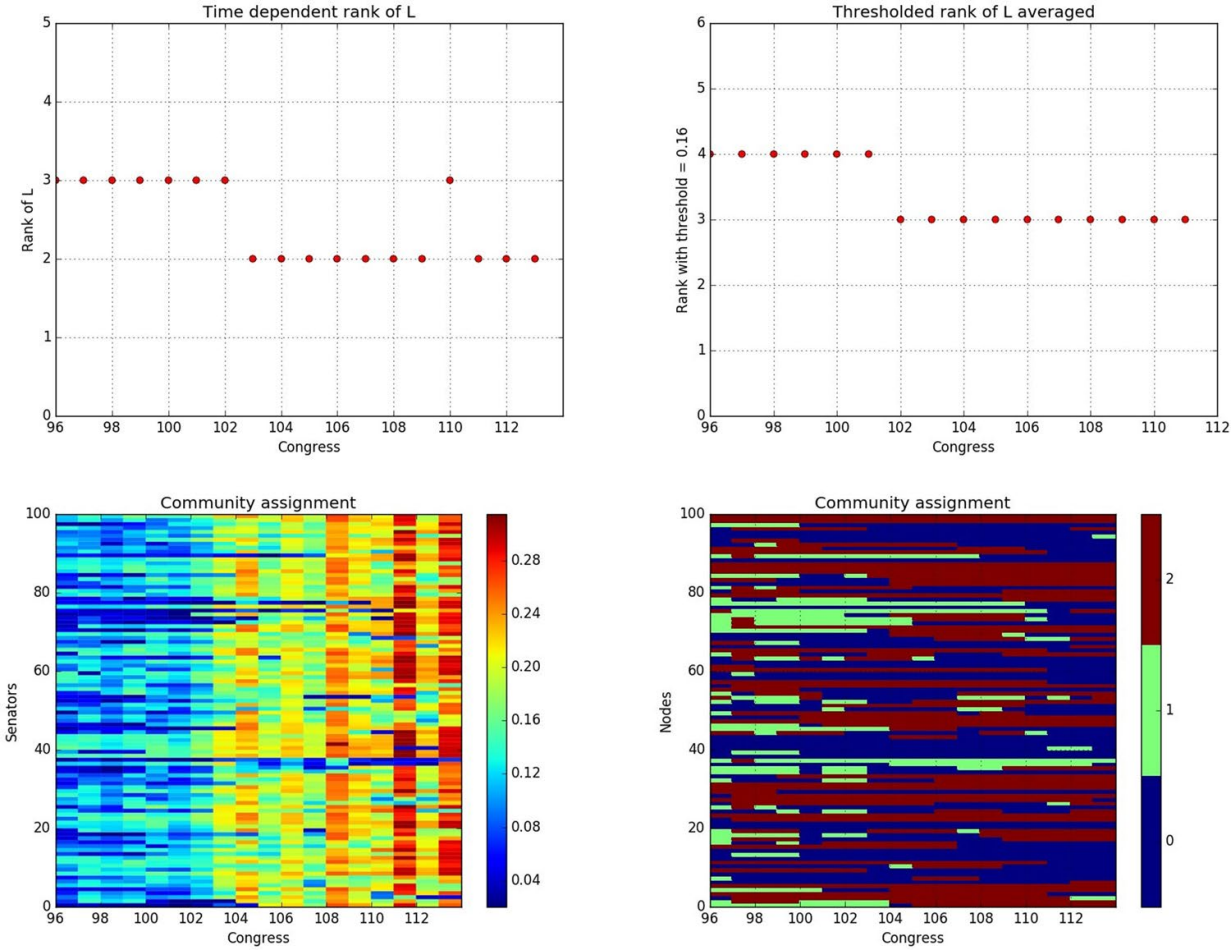
(**a**) Upper left panel, rank of recovered *L*(*t*) across time. (**b**) Upper right panel, *thresholded rank* of windows of size 2 around the phase transition epoch. (**c**) Lower left panel, *variation* of each node across Congresses. (**d**) Lower right panel, community membership based on individual *L*(*t*) across Congresses.

Based on the strategy discussed for selection of tuning parameters, we identify the optimal *α* = 0.4 $(\gamma =\frac{1}{\sqrt{N}}=0.1)$, based on which, we obtain the time dependent rank of *L*(*t*) and *thresholded rank* of the averaged *L*(*t*) over windows of length 2, as shown in panels (a) and (b) of Fig. 8. Based on the above results, we conclude that there is a phase transition that occurs in late 1992, when Congress 102 ended its tenure and 103 started its. Further, the identified phase transition occurs a bit earlier (end of 1992) than the one mentioned in^21,50^ (end of 1994). Note that the time dependent rank indicates three communities before the phase transition in late 1992, that correspond to the core Democrat Senate members, the core Republican ones, while the third one includes Senators exhibiting a higher degree of bipartisanship. For the stable period after 1993, the network structure coalesces to two core communities corresponding to the two parties. On the other hand, the averaged across Congresses community structure shows four communities before 1992, that can be categorized as the core Democrats, core Republicans, voting Democrat (most of the time) and voting Republican (most of the time), while the latter two coalesce to a weakly connected community that exhibits some degree of bipartisanship after 1993.

To obtain further insights from this analysis, we also compute for each node *i* the *sample standard deviation* of its connections with the other nodes for all votes undertaken during the tenure of a Congress and call it *variation*, which represents the polarity of the node:

8 $${V}_{i}(t):\,=\sqrt{\frac{1}{N-1}\,\sum _{j=1}^{N}\,{[{A}_{ij}(t)-\frac{1}{N}\sum _{k=1}^{N}{A}_{ik}(t)]}^{2}}$$

Hence, higher values of *V*_*i*_ indicate stronger agreement with the party vote, while lower values indicate a more bipartisan attitude for the node, since on certain votes they follow the party line and on other ones cross the aisle and follow with their political opponents. Hence, the community structure across all Congresses (before and after the phase transition point identified) are 3 communities. From panels (c) and (d) in Fig. 8, we can see clearly that the recovered time dependent community structure accurately coincides with the time dependent variation structure in terms of outliers.

**Figure 9:**
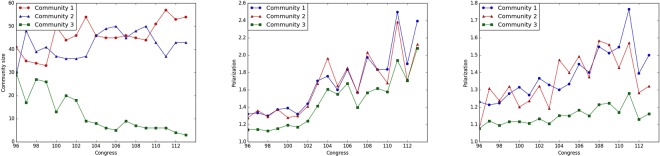
(**a**) Left panel, size of the three communities based on individual *L*(*t*) across Congress. (**b**) Middle panel, relative polarization of the three communities based on individual *L*(*t*) across Congress. (**c**) Right panel, relative polarization of the three communities based on the averaged ${\overline{L}}_{m}$ for the two stable periods.

Further, we plot in Fig. 9 the time dependent size and *relative polarization* (introduced in equation 7) for the community structure obtained based on individual low-rank matrices *L*(*t*), as well as that obtained from the two averaged $\overline{L}$ for the before 1992 and after 1993 periods. For community structure based on individual *L*(*t*), it can be seen that the size of the non-core community (the one exhibiting a bipartisan voting record) diminishes over time, while the relative polarization of all the three communities becomes higher. It is also worth noting that the relative polarization of the third community significantly increased and approached that of the other two over time. However, this result should not be over-interpreted, since the size of the latter community shrunk over time (note the variation of the respective nodes is still small (Fig. 8(c)) because of the small community size). On the other hand, the polarization of the third community recovered based on $\overline{L}$ is consistently low. For the first stable period, this indicates the success in capturing the core communities by the first two identified in the low-rank component, while for the second stable period, it is because the third community includes members from both the *polarized D* and *R* parties in each network snapshot, which induces the same amount of inter-community links as the intra-community ones, thus leading to low polarization. This is consistent with the results shown in Fig. 4, where *C*3 for the second stable period captures mainly the party flipping seats as further elaborated on in the ensuing discussion.

To further understand the community structure, in each stable period we manually assign party affiliations to each node by counting the ratio of each party affiliation by the following criteria. Denoting Republican Senators by *R* and Democrats by *D*, we assign nodes to these labels if over 75% of the time the corresponding party affiliation is *R* (*D*), otherwise we assign to a so-called mixed *M party*. The mixed *party* indicates that the corresponding Senate seat flips between parties over time, while the core *D*/*R* seats are stably held by one party over time. Table 4 provides a correspondence between party affiliations (*D*, *R*, and *M*) and the identified communities (*C*1, *C*2, and *C*3) based on averaged $\overline{L}$ for the two stable periods that cover Congress 96–102 and Congress 103–113, respectively. For each of the two stable periods, C1 captures the core “Democratic Party” seats, C2 captures the core “Republican Party” seats, and C3 captures the “Outliers”, in terms of voting similarities.

**Table 4:** Misclassification Table.

Party	Congress 96–102			Congress 103–113		
	C1	C2	C3	C1	C2	C3
D	29	0	10	33	0	1
R	0	29	10	0	29	3
M	3	0	19	4	4	26

**Figure 10:**
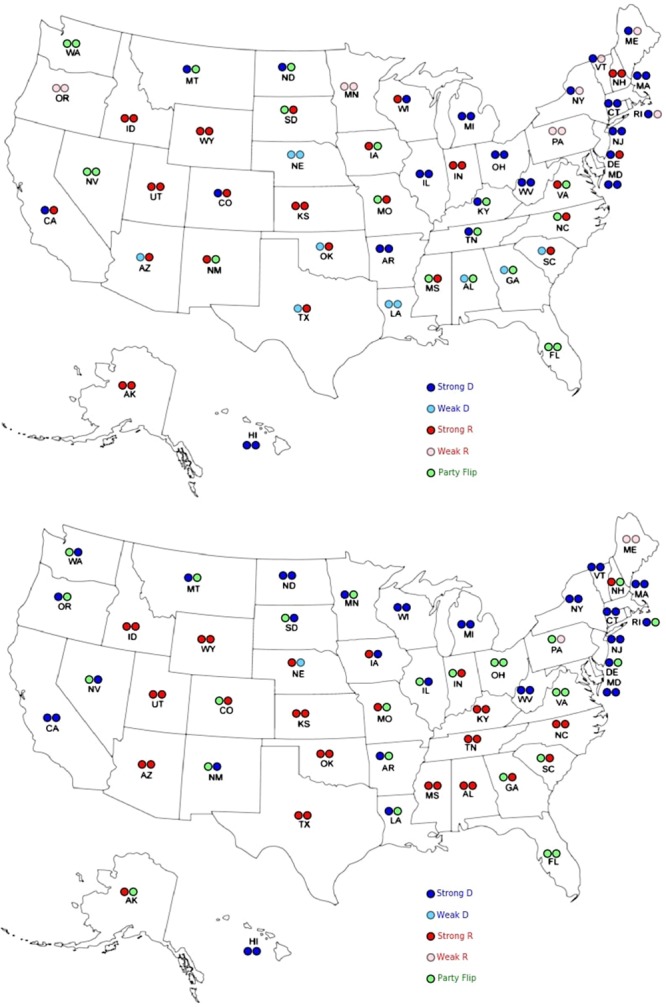
Clustering results based on averaged ${\overline{L}}_{m}$ over the two stable periods. (**a**) Upper panel, Congress 96–102. (**b**) Lower panel, Congress 103–113.

It is expected that nodes in the *M party* should be clustered in the third community (*C*3), which is consistent with the results in Table 4. Further, for both time ranges *C*1 basically captures the core members of *D* while *C*2 captures those of *R*. An interesting pattern that the analysis identifies is that almost all members of *C*3 for Congress 103–113 come from *M*, i.e. party flipping, while for Congress 96–102, more than half come from the two parties. This result agrees with what is shown in Fig. 9(a): namely, there are fewer outliers in Congress 103–113 compared to the previous time range. Finally, to gain further insights of where the third non-core community is more predominant, as shown in Fig. 10, we translate the results and findings of our analysis by laying them out on the US map, using the following notation for party identification purposes, which is in agreement with recent historical trends in the composition of Congress: *Strong D* for *C*1, *Weak D* for $D\cap C3$, *Strong R* for *C*2, *Weak R* for $R\cap C3$ and *Party Flip* for $M\cap C3$. Intuitively speaking, in each stable period, *Strong D*(*R*) represents those seats that consistently exhibit strong alignment with the core party members in *D*(*R*) in their voting behavior throughout the stable time period, even though some of the seats may not be stably held by the corresponding party (e.g. $M\cap C1(C2)$). Further, *Weak D*(*R*) indicates seats that consistently display bipartisan trends in voting behavior throughout the stable period although they are stably held by the *D*(*R*) party, and finally *Party Flip* represents those seats that, averaged over the stable time period, display bipartisan attitude, but mainly due to the fact that their holders lose the seat and are not re-elected (hence, the party flipping label).

## Concluding Remarks

The proposed methodology encompasses a number of network formation models that give rise to network community structure. As illustrated on a number of synthetic and real data examples, it is highly capable of identifying phase transition epochs, estimate accurately the number of communities and their membership. It is further computationally scalable, since obtaining the decomposition of the network adjacency matrices at each time point is a highly parallelizable step. Hence, the main computational bottleneck stems from the Singular Value Decomposition in obtaining the low-rank components *L*(*t*).

Further, note that the model can be further generalized and does not require a fixed low-rank component between phase transition epochs. The key requirement is that the community membership remains fixed as demonstrated through fixed rank, while the strength of their connections can fluctuate, as long as their average strengths over the length of the stable interval converges quickly. Hence, the proposed methodology would still be able to identify the transition epochs and extract the stable community structure provided that the length of the corresponding stable time intervals is adequate.

Finally, it would be of interest to couple the proposed strategy of identifying transition epochs, with more formal methods in change point analysis that come with statistical guarantees, as briefly outlined earlier on. One possibility is to extract the top eigenvectors from the adjacency matrices and then consider them as a multivariate time series. A possible challenge that requires careful study comes from the impact that the sparse and dense noise components considered may have on the extracted eigenvectors.

### Data availability

The data sets generated during and/or analyzed during the current study are available from the corresponding author on reasonable request.
